# Cell composition and expansion strategy can reduce the beneficial effect of AKT-inhibition on functionality of CD8^+^ T cells

**DOI:** 10.1007/s00262-020-02612-w

**Published:** 2020-06-05

**Authors:** Charlotte M. Mousset, Willemijn Hobo, Aafke de Ligt, Sjoerd Baardman, Nicolaas P. M. Schaap, Joop H. Jansen, Anniek B. van der Waart, Harry Dolstra

**Affiliations:** 1 Department of Laboratory Medicine, Laboratory of Hematology, Radboud Institute of Molecular Life Sciences, Radboud university medical center, Geert Grooteplein Zuid 8, 6525 GA Nijmegen, The Netherlands; 2 Department of Hematology, Radboud university medical center, Nijmegen, The Netherlands

**Keywords:** T cell, AKT, Memory, Polyfunctionality, Th1, Th2

## Abstract

AKT-inhibition is a promising approach to improve T cell therapies; however, its effect on CD4^+^ T cells is insufficiently explored. Previously, we and others showed that AKT-inhibition during ex vivo CD8^+^ T cell expansion facilitates the generation of polyfunctional T cells with stem cell memory-like traits. However, most therapeutic T cell products are generated from lymphocytes, containing CD4^+^ T cells that can affect CD8^+^ T cells dependent on the Th-subset. Here, we investigated the effect of AKT-inhibition on CD4^+^ T cells, during separate as well as total T cell expansions. Interestingly, ex vivo AKT-inhibition preserved the early memory phenotype of CD4^+^ T cells based on higher CD62L, CXCR4 and CCR7 expression. However, in the presence of AKT-inhibition, Th-differentiation was skewed toward more Th2-associated at the expense of Th1-associated cells. Importantly, the favorable effect of AKT-inhibition on the functionality of CD8^+^ T cells drastically diminished in the presence of CD4^+^ T cells. Moreover, also the expansion method influenced the effect of AKT-inhibition on CD8^+^ T cells. These findings indicate that the effect of AKT-inhibition on CD8^+^ T cells is dependent on cell composition and expansion strategy, where presence of CD4^+^ T cells as well as polyclonal stimulation impede the favorable effect of AKT-inhibition.

**Electronic supplementary material:**

The online version of this article (10.1007/s00262-020-02612-w) contains supplementary material, which is available to authorized users.

## Introduction

Adoptive transfer of tumor-reactive T lymphocytes has the potential to induce sustained clinical remission in patients with advanced cancer [1–3]. Clinical efficacy seems dependent amongst others on the infusion of tumor-reactive T cells with early memory-like characteristics [4–6]. Previously, we and others showed that transient pharmacological AKT-inhibition during ex vivo CD8^+^ T cell expansion that facilitates the generation of T cells with a stem cell memory (T_SCM_)-like phenotype [7–11]. Interestingly, AKT-inhibition during activation of CD8^+^ T cells allows proliferation, while preserving the early memory phenotype with high expression of CD62L, CCR7, CXCR4, CD27 and CD28 [7–12]. Furthermore, the gene expression profile of AKT-inhibited CD8^+^ T cells resembled T_SCM_ cells, which are known for their superior persistence in vivo [11, 13]*.* Importantly, AKT-inhibited CD8^+^ T cells showed increased expansion capacity upon recall, increased anti-tumor reactivity and enhanced polyfunctionality by co-producing IFNγ and IL2 [7–11]. This makes transient AKT-inhibition an interesting approach to improve adoptive T cell products, including ex vivo expanded tumor infiltrating lymphocytes (TILs), chimeric antigen receptor (CAR) T cells and T cell receptor (TCR)-transduced T cells [9, 12, 14, 15]. Currently, no clinical trials regarding AKT-inhibited cell therapies have been performed yet. Nevertheless, inhibiting the PI3K/AKT-pathway in cell therapy is currently used, as a Phase I clinical trial is recruiting multiple myeloma patients for the treatment with PI3K-inhibited BCMA CAR T cells (NCT03274219). However, most of these cell therapy products are generated from the total CD3^+^ T cell fraction or total PBMCs, containing also CD4^+^ T cells. Though generation of early memory CD4^+^ T cells could be beneficial for a long-term anti-tumor effect, they can influence CD8^+^ T cell expansion and function depending on their helper subset [16–19]. Therefore, we investigated the effect of transient in vitro AKT-inhibition during CD4^+^ T cell expansion, focusing on memory and Th-subset differentiation, and its effect on CD8^+^ T cell functionality.

Like CD8^+^ T cells, naive CD4^+^ T (T_N_) cells differentiate into T_SCM_, central memory T (T_CM_) cells, effector memory T (T_EM_) cells and effector T (T_EFF_) cells [20]. Besides effector/memory differentiation, CD4^+^ T cells also acquire differential functional traits. The most prominent populations are CD4^+^ Th1, Th2, Th17 and Treg cells. Discrimination is mainly based on cytokine production profiles, in combination with expression of extracellular markers and transcription factors. The different CD4^+^ T cell subsets have distinctive helper functions, with Th1 cells being described as the most potent to support anti-tumor immunity. Th1 cells produce IFNγ and IL2, thereby promoting priming and expansion of CD8^+^ T cells, and recruitment of NK cells and type I macrophages [21, 22]. In contrast, presence of Tregs is unfavorable for anti-tumor immunity, where high Treg:CD8 ratios are correlated with poor patient survival [23, 24]. Th2 and Th17 cells could either promote or reduce anti-tumor effect, depending several factors, including the immune population in the tumor microenvironment [25–30]. Moreover, as the CD4^+^ T helper subset may influence CD8^+^ T cell functionality when cultured together, it is essential to determine the effect of transient AKT-inhibition during a combined ex vivo expansion.

Previous studies showed that the PI3K/AKT-pathway plays a role in skewing differentiation toward CD4^+^ T helper subsets. AKT signaling can support Th1 and Th17 differentiation via mTORC1, while Th2 differentiation is stimulated via PI3K and mTORC2 [31–34]. Furthermore, inhibition of AKT and mTORC1 can cause induction of Tregs [35, 36]. Together, this indicates that inhibition of AKT during expansion of CD4^+^ T cells might stimulate Th2 and Treg differentiation, at the expense of Th1 and Th17. Therefore, ex vivo AKT-inhibition during the generation of T cell therapy might negatively influence CD4^+^ and CD8^+^ T cell function when applied to total CD3^+^ T cells.

In this study, we investigated the effect of Akt-inhibitor VIII (AktiVIII) and GDC-0068 (GDC) on CD4^+^ T cell effector/memory and functional helper subset differentiation. AktiVIII and GDC have an allosteric or adenosine triphosphate (ATP)-competitive mode of action, respectively, and previously showed to be the most promising AKT-inhibitors for the generation of T_SCM_-like CD8^+^ T cells during T cell expansion by dendritic cells (DCs) [11]. Here, we show that next to CD8^+^ T cells, both AKT-inhibitors preserved memory differentiation in CD4^+^ T cells reflected by higher expression of CD62L, CXCR4 and CCR7. However, Th-subset skewing was altered by AKT-inhibition, with less Th1 and more Th2-associated cells compared to control cultures. Importantly, the favorable effect of AKT-inhibition on the functionality of CD8^+^ T cells drastically diminished in the presence of CD4^+^ T cells. Moreover, the method of expansion did also influence rechallenge capacity, and the effect of AKT-inhibition on CD8^+^ T cells degranulation and polyfunctionality. These findings indicate that the effect of AKT-inhibition on CD8^+^ T cells is dependent on cell composition and expansion strategy, where presence of CD4^+^ T cells as well as polyclonal stimulation impede the favorable effect of AKT-inhibition on CD8^+^ T cells.

## Materials and methods

### Cell material and isolation

PBMCs were isolated from buffy coats of healthy donors (Sanquin Blood Supply Foundation) using Ficoll-paque™ PLUS (cat#17-1440-03, Sigma-Aldrich) density gradient centrifugation. CD14^+^ monocytes were isolated from fresh material using anti-CD14 immunomagnetic beads (cat#130-050-201, Miltenyi Biotec). Peripheral blood lymphocytes were cryopreserved in medium containing 10% (cat#102,952, DMSO, Merck Millipore) and 10% serum using a Mr.Frosty™ (Thermofisher). T cells were isolated from fresh or frozen material via magnetic bead isolation using the EasySep™ Human Naive CD4^+^ T Cell Isolation Kit (cat#19555, Stemcell Technologies) or CD3, CD4 or CD8 microbeads (cat#130-050-101, cat#130-045-101, and cat#130-045-201, Miltenyi Biotec). The obtained purity was on average 95% (ranging from 91 to 99%) and CD3, CD4 and CD8 bead-isolated T cells contained on average 52% T_N_ cells (ranging from 27 to 83%). Cell numbers were based on trypan blue counting and flow cytometric quantification of relative and absolute cell numbers.

### T cell cultures

To generate mature DCs, monocytes were cultured for 2–3 days in manufactory pre-tested X-VIVO™ 15 medium (cat#BE02-061Q or cat#BE02-060Q, Lonza) supplemented with 2% human serum (HS, Sanquin Blood Supply Foundation, not pre-tested for assay performance), 500 IU/ml IL4 and 800 IU/ml GM-CSF (cat#11340045 and cat#11343125, ImmunoTools). Subsequently, cells were re-plated at 0.5 × 10^6^/ml in fresh X-VIVO™ 15 containing 2% HS, 500 IU/ml IL4 and 800 IU/ml GM-CSF for another 3–4 days. Next, immature DCs were maturated for 2 days by adding X-VIVO™ 15 containing 2% HS and cytokines at a final concentration of 5 ng/ml IL1β, 15 ng/ml IL6, 20 ng/ml TNFα (cat#11340015, cat#11340064, cat#11343015, all ImmunoTools) and 1 µg/ml PGE2 (Prostin E2®, Pfizer).

CD3^+^ T cells, CD4^+^ T cells, CD4^+^ T_N_ or CD8^+^ T cells were stimulated with allogeneic DCs at a 1:10 DC:T cell ratio or with CD3/CD28 Dynabeads (cat#11131D, ThermoFisher) at a 1:1 bead: T cell ratio for 10 days in IMDM (cat#12440053, Thermofisher), supplemented with 10% HS, 50 IU/ml IL2 (Proleukin®, Chiron), 5 ng/ml IL7 and 5 ng/ml IL15 (cat#11340075 and cat#11340155, both ImmunoTools). When indicated, Akt-inhibitor VIII (cat#A6730, Sigma) or GDC-0068 (cat#HY-15186, MedChemExpress), dissolved in DMSO, was added. Control conditions were supplemented with corresponding concentrations of DMSO (≤ 0.1% in all experiments). Half of the culture volume was refreshed with medium plus cytokines and AKT-inhibitor or DMSO every 2–3 days. Cells were rechallenged with DCs or CD3/CD28 beads on day 10 and cultured for another 7 days without AKT-inhibitor or DMSO. To evaluate cytokine production profiles on day 10 or 17, T cells were restimulated overnight with DCs (1:10 DC:T cell ratio) or 1 µg/ml PMA plus 40 µg/ml ionomycin (cat# P1585 and cat#I0634, both Sigma), in the presence of Brefeldin A (Golgiplug, cat#555029, BD Biosciences) or Monensin (Golgistop, cat#554724 BD Biosciences), in absence of AKT-inhibitor or DMSO.

### Flow cytometry

Immunophenotypical analysis was performed by flow cytometry. Cells were washed with PBS (cat#3623130, Braun) containing 0.5% bovine serum albumin (BSA, cat#A9647, Sigma) followed by extracellular staining for 30 min at 4 °C in the dark with antibodies against CD3 (UCHT1, Biolegend), CD4 (SK3, Biolegend), CD8 (RPA-T8, Biolegend), CD45RO (UCHL1, Beckman Coulter) and Fixable Viability Dye eFluor780 (cat#65-0865-14, ThermoFisher). Additionally, for phenotyping the following antibodies were used: CCR7 (G04H7, Biolegend), CD62L (DREG56, Biolegend), CXCR4 (12G5, Biolegend), CXCR3 (G025H7, Biolegend), CCR4 (L291H4, Biolegend), CCR6 (G034E3, Biolegend) and CD25 (B1.49.9, Beckman Coulter). For intracellular transcription factor staining, cells were fixated and permeabilized with Fixation/Permeabilization Concentrate and Diluent (cat#00-5123-43, and cat#005223-56, both eBioscience) and stained in Permeabilization Buffer (cat#00-8333-56, eBioscience) with antibodies for T-bet (4B10, eBioscience), GATA3 (16E10A23, Biolegend), RORγT (AFKJS-9, eBioscience) and FOXP3 (3G3, eBioscience). Intracellular staining of CD4^+^ T cell cytokines was performed upon fixation and permeabilization with Fixation/Permeabilization Concentrate and Diluent after overnight restimulation in presence of Brefeldin A to analyze IFNγ (B27, BD Biosciences), IL4 (8D4-8, BD Biosciences) and IL17 (BL168, Biolegend), or Monensin to analyze IL10 (JES3-9D7, BD Biosciences). CD8^+^ T cell polyfunctionality was analyzed after overnight stimulation in presence of Brefeldin A and CD107a (H4A3, Biolegend) after which cells were fixated with 4% paraformaldehyde (cat#P6148, BOOM) for 10 min at RT in the dark, followed by permeabilization with 0.1% saponin (cat#47036, Sigma) buffer containing 10% FCS (Integro B.V.) for 10 min at RT. Staining of IFNγ, IL2 (5344.111, BD Biosciences) and TNFα (MAb11, BD Biosciences) was performed in 0.1% saponin buffer for 30 min at 4 °C in the dark. After staining, cell data acquisition was performed on the Gallios flow cytometer (Beckman Coulter). Flow cytometry data were analyzed with Kaluza software (Beckman Coulter, version 1.5a and 2.1). Delta median fluorescence intensity (ΔMFI) was calculated by subtracting the MFI of the marker of interest with the MFI of the background fluorescence within the population of interest. Polyfunctionality of CD8^+^ T cells was visualized using SPICE version 5.3 (NIH/NIAID, MD, USA).

### Additional information regarding Minimal Information About T cell Assays (MIATA)

This paper is MIATA-compliant. This study was conducted in a laboratory that operates under exploratory research principles. Experiments are performed in general research investigative assays according to investigative protocols. Raw data can be provided per request. The MIATA checklist is provided as Supplementary information.

### Statistical analysis

Statistical analysis was performed with Prism software (Graphpad Software Inc., version 5.03) using a Student’s *T*-test or one-way ANOVA, followed by a Bonferroni post-hoc test as indicated in the figure legends.

## Results

### AKT-inhibition preserves memory differentiation of CD4^+^ T cells

**Fig. 1:**
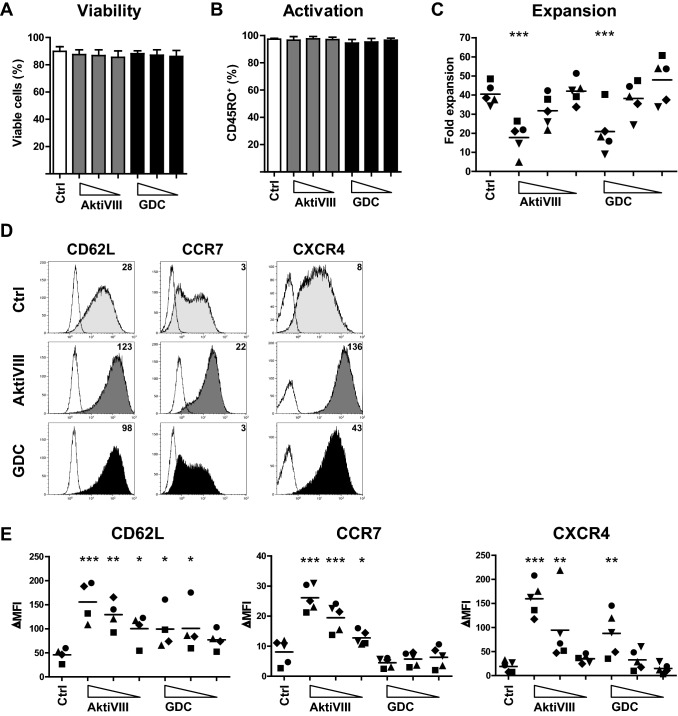
AKT-inhibition preserves CD4^+^ T cell differentiation. CD4^+^ T_N_ cells were stimulated with allogeneic DC in presence of DMSO (Ctrl), AktiVIII (18 µM, 12 µM, 6 µM) or GDC (10 µM, 5 µM, 2.5 µM) for 10 days. **a** Percentage viable and **b** activated (CD45RO^+^) CD4^+^ T cells. Mean + SEM for 5 independent donors is shown. **c** Fold expansion of CD4^+^ T cells between day 0–10. Individual donors are depicted with unique symbols. **d**–**e** Surface expression of CD62L, CCR7 and CXCR4 on CD3^+^CD4^+^CD45RO^+^ T cells of (**d**) 1 representative donor (filled) with corresponding background staining (open), where numbers represent ΔMFI, and **e** expression overview for 4–5 independent donors. Statistical analysis was performed using Repeated Measures ANOVA followed by Bonferroni’s Multiple Comparison Test, comparing AKT-inhibited with Ctrl T cells. ***p < 0.001; **p < 0.01; *p < 0.05

We previously showed that T_SCM_-like CD8^+^ T cells can be generated by transient AKT-inhibition during ex vivo T cell activation and expansion by allo-antigen or minor histocompatibility antigen-presenting DCs [7, 11]. To study the effect of AKT-inhibition on effector memory differentiation of CD4^+^ T cells, we stimulated CD4 T_N_ cells with allogeneic DCs in combination with increasing dosages of AKT-inhibitor. First, DMSO drug solvent effects were excluded, as viability, activation, expansion and differentiation of CD4^+^ T cells were not affected (Supplementary Fig. 1). Notably, increasing dosages of AKT-inhibitor did not affect viability (Fig. 1a) nor activation (Fig. 1b), though a dose-dependent effect on expansion was observed (Fig. 1c). CD4^+^ T cell expansion reduced from 40-fold in control conditions to 20-fold at the highest dose of AktiVIII (18 µM) and GDC (10 µM) during the 10-day culture period. Importantly, the naive-associated marker CD62L was dose-dependently retained on CD4^+^ T cells cultured with AKT-inhibitors (Fig. 1d, e). In addition, CCR7 and CXCR4 were preserved on AktiVIII-treated CD4^+^ T cells, while these effects were less prominent for GDC-treated CD4^+^ T cells. Especially, CCR7 could not be preserved by this ATP-competitive AKT-inhibitor. Overall, ex vivo AKT-inhibition preserved memory differentiation of CD4^+^ T_N_ cells upon stimulation in a dose-dependent manner, with the strongest effects shown for the allosteric inhibitor AktiVIII.

### AKT-inhibition promotes Th2 skewing at the expense of Th1 cells

**Fig. 2:**
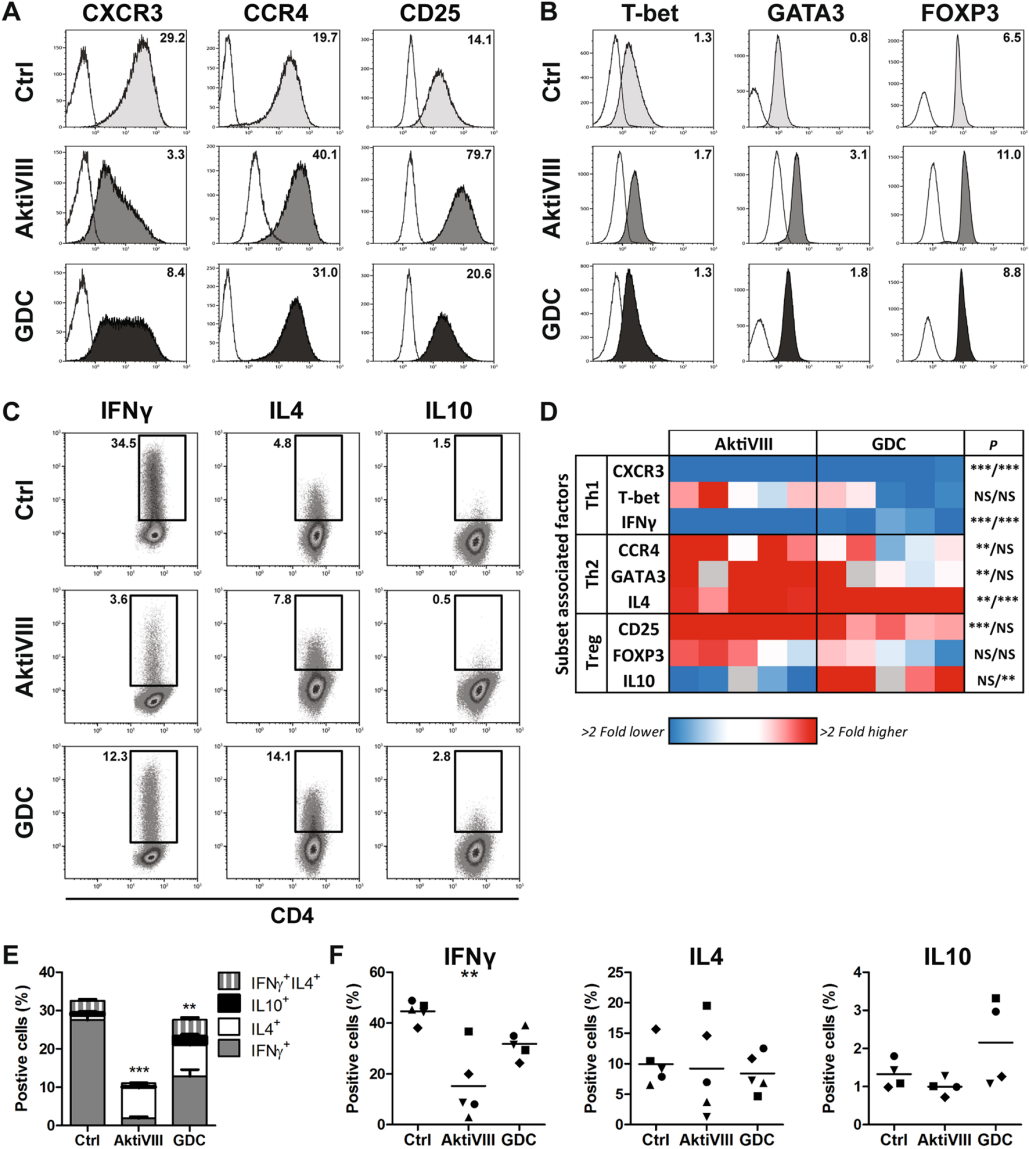
AKT-inhibition modulates CD4^+^ T cell skewing of Th1 and Th2. CD4^+^ T_N_ cells were stimulated with allogeneic DCs in presence of DMSO (Ctrl), AktiVIII (18 µM) or GDC (10 µM) for 10 days. **a** Surface expression (filled) or **b** intracellular transcription factor expression (filled) and corresponding background staining (open) on/in CD3^+^CD4^+^CD45RO^+^ T cells. Data shown for 1 representative donor. Numbers represent ΔMFI. **c** Cytokine production of CD3^+^CD4^+^CD45RO^+^ T cells of 1 representative donor after overnight restimulation with allogeneic DCs in absence of AKT-inhibitors. Numbers show percentages cytokine-producing cells. **d** Heatmap showing fold change compared to Ctrl condition of different Th cell-associated parameters for 4–5 independent donors. Grey = no data. **e** Total cytokine production and composition of CD3^+^CD4^+^CD45RO^+^ T cells for 4 independent donors, mean + SEM. IL10 production was analyzed in separate tube and is therefore not corrected for co-production. **f** Percentage IFNγ, IL4 or IL10 producing cells on day 18 upon overnight restimulation with allogeneic DCs after 1 week of rechallenge in absence of AKT-inhibitors. Data are shown for 4–5 independent donors. Individual donors are depicted with unique symbols. **d**–**f** Cytokine production is shown corrected for non-stimulated conditions. Statistical analysis was performed using Repeated Measures ANOVA followed by Bonferroni’s Multiple Comparison Test, comparing AKT-inhibited with Ctrl T cells, with in **e** performed on the total cytokine production. ***p < 0.001, **p < 0.01, *p < 0.05, *NS* non-significant

Besides effector memory differentiation, CD4^+^ T cell develop toward T helper subsets with distinct effector functions upon activation of naive T cells. Therefore, the effect of AKT-inhibition on subset skewing toward Th1, Th2, Th17 and Tregs was evaluated based on extracellular phenotype, intracellular expression of transcription factors and cytokine secretion profiles. The Th17-associated markers (CCR6, RORγT and IL17) were not found upon activation of CD4^+^ T_N_ cells and therefore not further evaluated in this assay. Remarkably, while the Th1 population was most dominant in control cultures, extracellular expression of the Th1-associated marker CXCR3 was lower on AKT-inhibited CD4^+^ T cells (Fig. 2a, d; Supplementary Fig. 2A). On the contrary, Th2-associated CCR4 was significantly higher expressed on AktiVIII-cultured cells (Fig. 2a, d and Supplementary Fig. 2A). Effects on transcription factor expression were less pronounced, with only significant higher levels of Th2-associated GATA3 in AktiVIII cultured cells (Fig. 2b, d and Supplementary Fig. 2B). Additionally, cytokine secretion profiles were analyzed after overnight restimulation with allogeneic DCs. AKT-inhibited cultures showed significantly lower percentages of IFNγ-producing T cells, confirming the inhibitory effect of AKT-inhibition on Th1 differentiation (Fig. 2c, d and Supplementary Fig. 2C). Again, skewing toward the Th2 subset was observed as reflected by the increased percentage of AKT-inhibited T cells producing IL4. Although the percentage of IL10-producing cells was low (≤ 3%), a small increase in this Treg-associated cytokine was observed for GDC-cultured conditions. Together, these data indicate that both AKT-inhibitors AktiVIII and GDC markedly skew the acquisition of CD4^+^ effector functions toward Th2 traits and less toward Th1 cell differentiation. Moreover, the total proportion of cytokine producing CD4^+^ T cells was significantly lower in AKT-inhibitor-treated cultures (Fig. 2e). Notably, when cells were released from their transient AKT-inhibition and cultured for an additional week, the negative effect on Th1 skewing was sustained, as IFNγ-production remained significantly lower in AktiVIII-treated cultures (Fig. 2f). Together, these data show that in vitro AKT-inhibition promotes CD4^+^ Th-subset skewing toward Th2-associated cells while diminishing Th1 skewing.

### AKT-inhibition changes CD4^+^ T cell skewing depending on cell composition

**Fig. 3:**
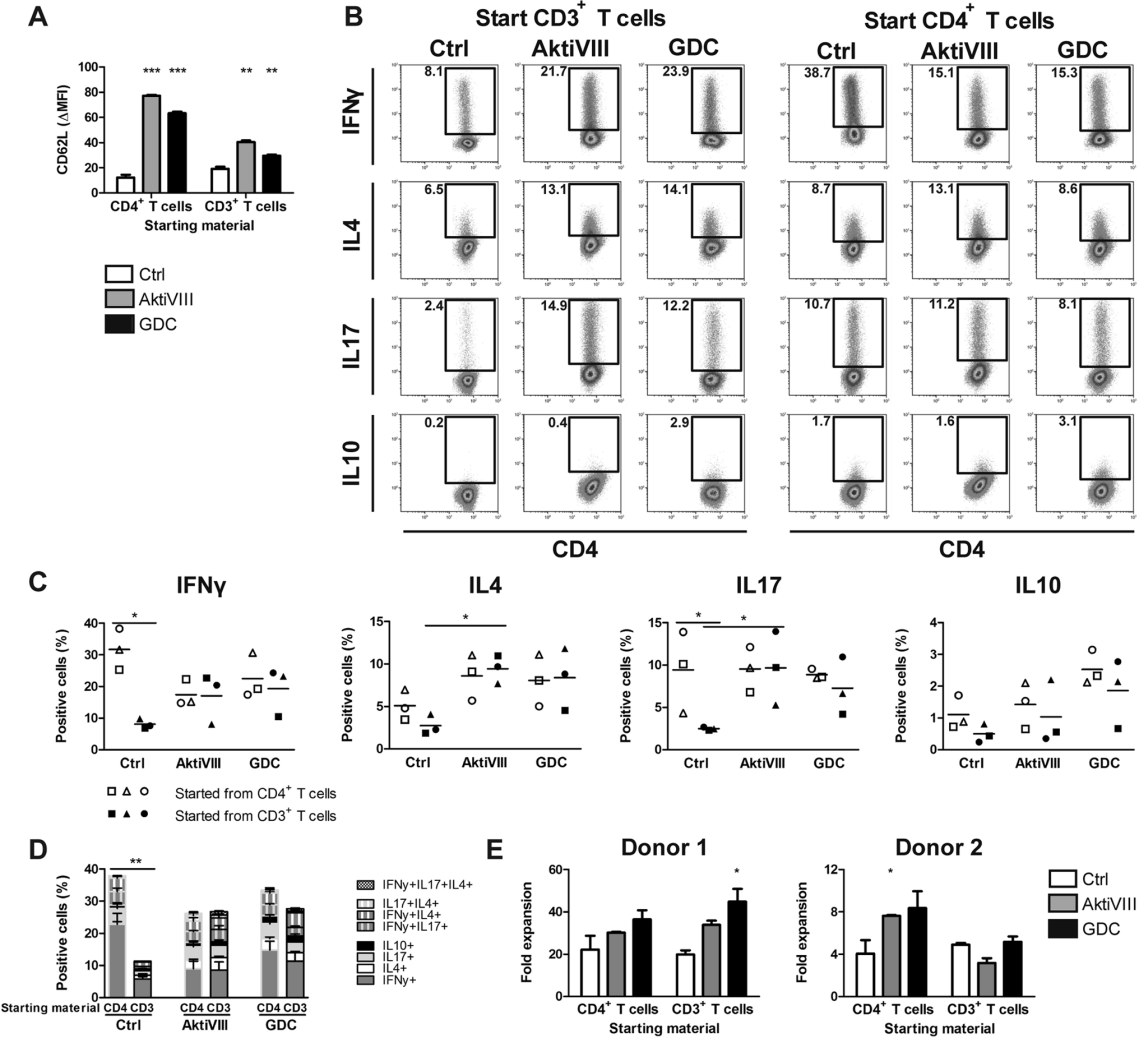
AKT-inhibition does not impede Th1 skewing of CD4^+^ T cells in cultures with total CD3^+^ T cells. Total CD3^+^ T cells or CD4^+^ T cells were stimulated with allogeneic DCs in presence of DMSO (Ctrl), AktiVIII (18 µM) or GDC (10 µM) for 10 days. **a** CD62L expression (ΔMFI) of CD3^+^CD4^+^CD45RO^+^ T cells in CD3^+^ or CD4^+^ T cell cultures. Mean + SD (*n* = 2) shown for 1 representative out of 3 donors. **b**–**d** Intracellular cytokine staining after O/N restimulation with DCs in absence of AKT-inhibitors. **b** Dotplots of 1 representative donor, numbers represent percentage cytokine producing cells. **c** Percentages IFNγ-, IL4-, IL17- and IL10-producing cells within the CD4^+^ T cell population, in CD4^+^ (open symbols) or CD3^+^ (closed symbols) T cell cultures for 3 independent donors, corrected for non-stimulated conditions. Individual donors are depicted with unique symbols. **d** Total cytokine production and composition of CD3^+^CD4^+^CD45RO^+^ T cells, corrected for non-stimulated conditions. Mean + SEM of 3 independent donors. IL10 production was analyzed in separate tube and therefore not corrected for co-production. Statistical analysis was performed using **a** one-way ANOVA and **c**–**d** Repeated Measures ANOVA followed by Bonferroni’s Multiple Comparison Test, comparing AKT-inhibited with Ctrl T cells, and cell composition within Ctrl or AKT-inhibited condition. ***p < 0.001, **p < 0.01, *p < 0.05

As manufacturing of most adoptive T cell products starts with total CD3^+^ T cells, containing total CD4^+^ instead of CD4^+^ T_N_ cells, we investigated the effect of AKT-inhibition on CD4^+^ T cell differentiation when starting with either total CD3^+^ or CD4^+^ T cell cultures. Similar to starting from CD4^+^ T_N_ cells, preserved memory differentiation in AKT-inhibited CD4^+^ T cells was observed (Fig. 3a and Supplementary Fig. 3). The cell composition of CD3^+^ T cell cultures consisted of a CD4:CD8 ratio of 2.0 ± 0.5, which shifted to a more balanced culture with a ratio of 0.9 ± 0.2 on day 10. The addition of AKT-inhibition did not affect this distribution. Interestingly, when both CD4^+^ and CD8^+^ T cells were present in the culture (started from CD3^+^ T cells), fewer cytokine-secreting CD4^+^ T cells were observed compared to cultures started from CD4^+^ T cells alone (Fig. 3b–d). Here, 38% of CD4^+^ T cells in single cultures were capable of secreting either IFNγ, IL4, TNFα and/or IL10 while in CD3^+^ T cells-derived cultures only 11% cytokine-secreting CD4^+^ T cells were observed. When AKT-inhibition was added to these cultures, secretion of the Th-associated cytokines was similar between inhibited cultures, independent of the cell composition. Compared to their controls, AKT-inhibition impeded the proportion of CD4^+^ T cells producing IFNγ when cultures were started with CD4^+^ T cells, though an increase was observed compared to controls of CD3^+^ T cell cultures (Fig. 3b, c). Moreover, also negative effects on CXCR3 and T-bet expression were less pronounced and more variable in total CD3^+^ T cell cultures compared to cultures started with solely CD4^+^ T cells (Supplementary Fig. 4). Nevertheless, independent of the cell composition, skewing toward Th2 cells was observed upon AKT-inhibition (Fig. 3b, c and Supplementary Fig. 4). Together, AKT-inhibition resulted in a decreased Th1-associated IFNγ/Th2-associated IL4 ratio (CD4^+^ T cell cultures: from 6.6 ± 1.5 in control conditions to 2.1 ± 0.5 and 2.9 ± 0.5 in AktiVIII and GDC-treated cultures, respectively, both *p* < 0.001; CD3^+^ T cell cultures: from 3.2 ± 0.6 in control conditions to 1.7 ± 0.5 and 2.3 ± 0.3 in AktiVIII and GDC-treated cultures, respectively). Moreover, IL17-producing CD4^+^ T cells could be detected when starting cultures with either total CD3^+^ or CD4^+^ T cells, with increased IL17-producing CD4^+^ T cells upon AKT-inhibition compared to control CD3^+^ T cells (Fig. 3b, c). Lastly, IL10-production was again low (≤ 3%) in these cultures, with no significant effect on IL10-producing cells upon AKT-inhibition. Furthermore, cell composition did not affect the expansion capacity of the cultured cells (Fig. 3e). Nevertheless, upon antigen recall in the absence of inhibitors a mild increase in expansion capacity of AKT-inhibited CD4^+^ T cells as was observed. Collectively, these data show that cell composition determine CD4^+^ T helper skewing and the effect of AKT-inhibition.

**Fig. 4:**
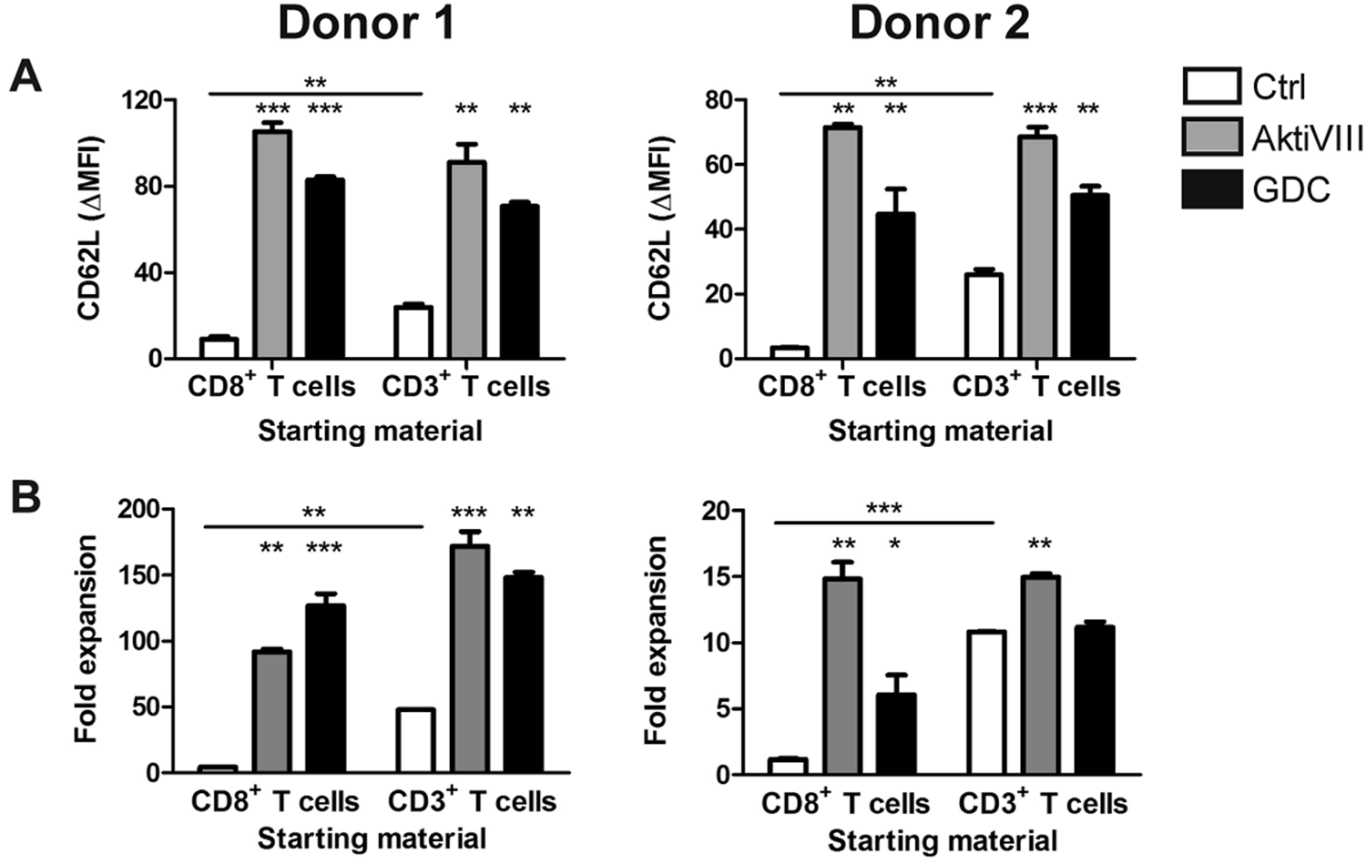
AKT-inhibition preserves early memory CD8^+^ T cell differentiation and improves rechallenge capacity of CD8^+^ T cells. Total CD3^+^ T cells or CD8^+^ T cells were stimulated with allogeneic DCs in presence of DMSO (Ctrl), AktiVIII (18 µM) or GDC (10 µM) for 10 days. **a** CD62L expression on CD3^+^CD8^+^CD45RO^+^ T cells for 2 independent donors, ΔMFI, Mean + SD, *n* = 2. **b** Fold expansion between day 10 and 17 upon rechallenge with DCs in absence of AKT-inhibitors, 2 independent donors, mean + SD, *n* = 2. Statistical analysis was performed using one-way ANOVA followed by Bonferroni’s Multiple Comparison Test, comparing AKT-inhibited with Ctrl T cells within each starting population. Ctrl conditions were compared with a two-tailed unpaired *T* test. ***p < 0.001, **p < 0.01, *p < 0.05

### Cell composition and expansion strategy affect AKT-inhibition of CD8^+^ T cells

As changes in the CD4^+^ Th-skewing could affect CD8^+^ T cell functionality, which is pivotal for effective anti-tumor efficacy, we analyzed the effect of AKT-inhibition on CD8^+^ T cell functionality in presence and absence of CD4^+^ T cells. First, we confirmed the preserved early memory phenotype of CD8^+^ T cells in AktiVIII and GDC-cultured conditions when starting with total CD3^+^ or CD8^+^ T cells based on CD62L expression (Fig. 4a). Notably, in (non-AKT-inhibited) control conditions the presence of CD4^+^ T cells reduced CD8^+^ T cell differentiation, shown by higher CD62L expression compared to cultures with CD8^+^ T cells alone. Furthermore, in control conditions the presence of CD4^+^ T cells also improved the rechallenge expansion capacity of control CD8^+^ T cells upon antigen recall as was expected (Fig. 4b). Importantly, transient AKT-inhibition provided significantly enhanced CD8^+^ T cell expansion upon rechallenge, though effects were less pronounced when CD4^+^ T cells were present in the starting material. To that end, we observed preserved memory differentiation of AKT-inhibited CD8^+^ T cells, though cell composition did affect memory phenotype and rechallenge expansion capacity.

**Fig. 5:**
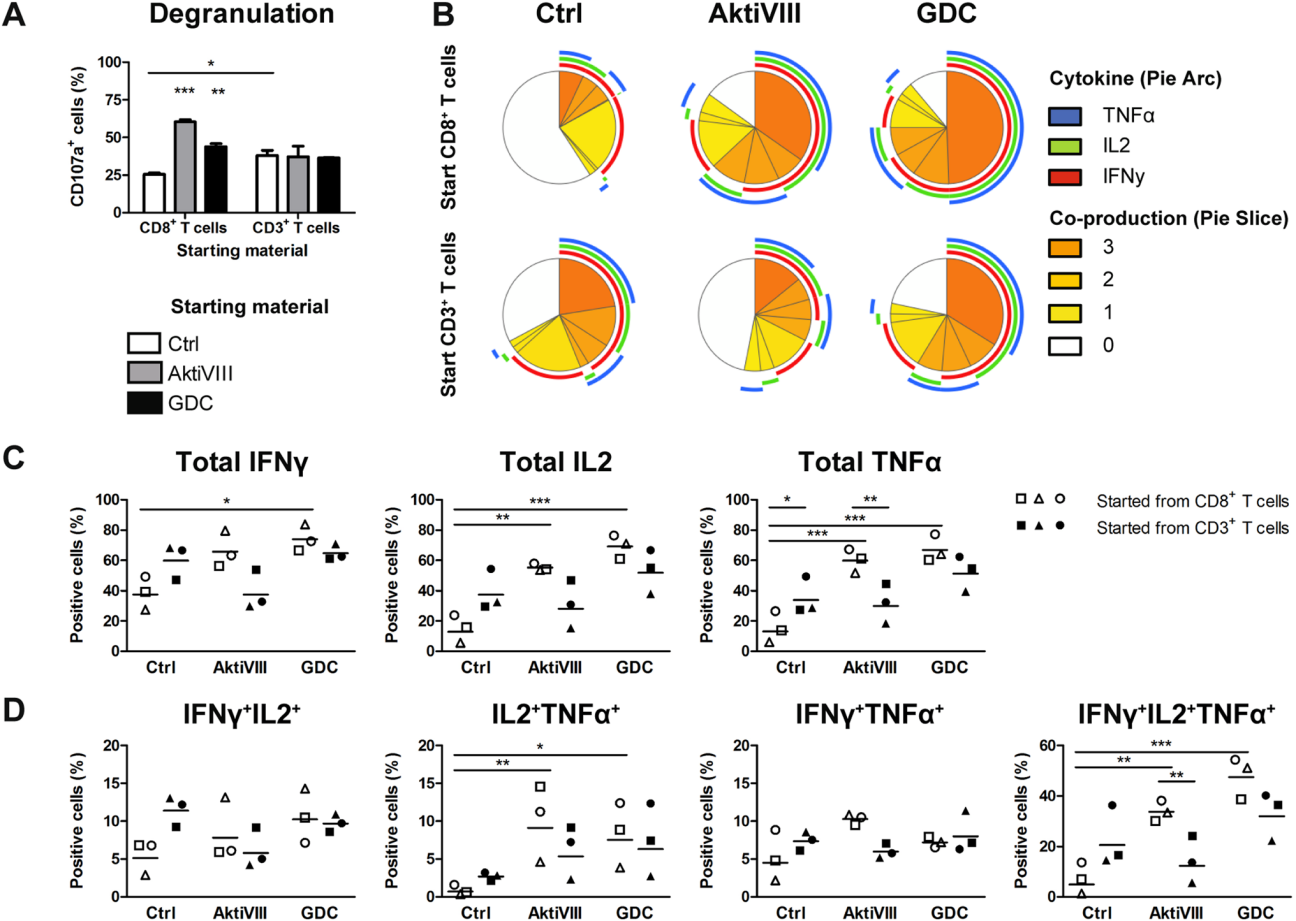
AKT-inhibition of allogeneic DCs expanded CD8^+^ T cells enhances polyfunctionality in absence of CD4^+^ T cells. Total CD3^+^ T cells or CD8^+^ T cells were stimulated with allogeneic DCs in presence of DMSO (Ctrl), AktiVIII (18 µM) or GDC (10uM) for 10 days. Cells were restimulated in absence of AKT-inhibition for 7 days. Degranulation and cytokine production were analyzed after O/N culture. **a** Percentages CD107a^+^CD3^+^CD8^+^ T cells, mean + SEM of 3 independent donors. **b**–**d** IFNγ-, IL2-, and TNFα-producing CD3^+^CD8^+^ T cells, **b** analyzed using SPICE software: the pie arcs depict the proportion of cells that produces a specific cytokine and pie slices the proportion of cells co-producing 0 to 3 different cytokines. **c** Total IFNγ-, IL2-, TNFα-, and **d** double and triple-producing cells within the CD8^+^ T cell population in CD8^+^ (open symbols) or CD3^+^ (closed symbols) T cell cultures. Statistical analysis was performed using Repeated Measures ANOVA followed by Bonferroni’s Multiple Comparison Test, comparing AKT-inhibited with Ctrl T cells within each starting population, and cell composition within Ctrl or AKT-inhibited condition. ***p < 0.001, **p < 0.01, *p < 0.05

As killing capacity and cytokine production of CD8^+^ T cells is pivotal for their function in anti-tumor immunity, we further analyzed CD8^+^ T cell functionality on degranulation and polyfunctional effector cytokine production after one week of rechallenge in absence of AKT-inhibitors. AKT-inhibited CD8^+^ T cells showed an improved degranulation compared to controls, but only when the expansion was performed in the absence of CD4^+^ T cells (Fig. 5a). Moreover, we observed an enhanced production of IFNγ, IL2 and TNFα by allogeneic-DC expanded AKT-inhibited CD8^+^ T cells (Fig. 5b–d and Supplementary Fig. 5). In addition, intracellular analysis showed that these effector cytokines were secreted by the same cells, illustrating polyfunctionality. However, the (polyfunctional) cytokine production of CD8^+^ T cells was dependent on cell composition, and only enhanced in AKT-inhibited cultures when starting with CD8^+^ T cells alone. Altogether, these data demonstrate that the beneficial effect of AKT-inhibition on the degranulation and polyfunctionality of CD8^+^ T cells is dependent on the composition of the cell starting material, where even though presence of CD4^+^ T cells in general improved (control) cultures, in these cultures they impede the favorable effect of AKT-inhibition on CD8^+^ T cells.

**Fig. 6:**
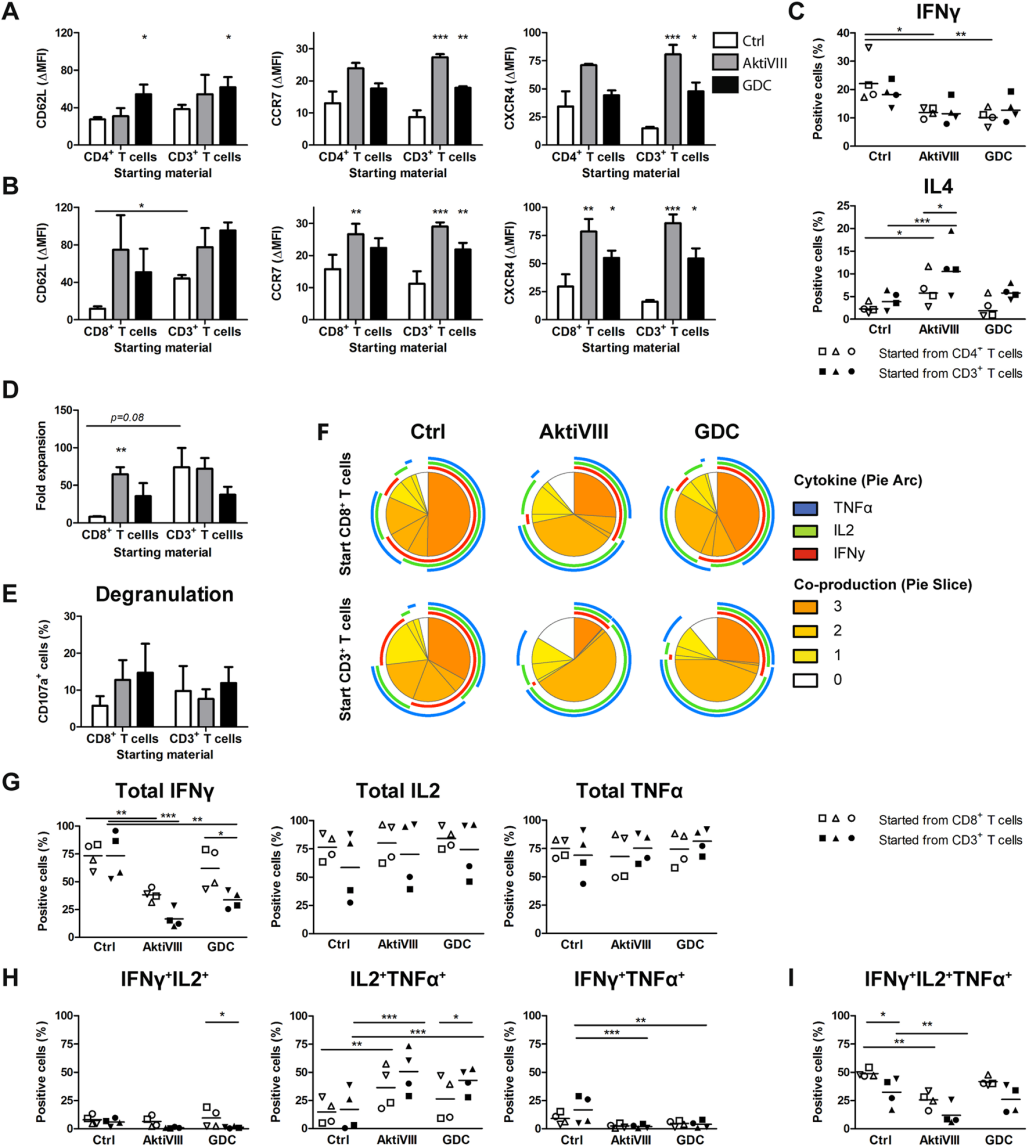
AKT-inhibition in polyclonal stimulated T cells induces early memory T cells, but affects Th1 and Th2 skewing and polyfunctionality of CD8+ T cells. Total CD4^+^ T cells, CD8^+^ T cells, or CD3^+^ T cells were stimulated with CD3/CD28 Dynabeads in presence of DMSO (Ctrl), AktiVIII (18 µM) or GDC (20 µM) for 10 days. **a**, **b** CD62L, CCR7 and CXCR4 expression on (**a**) CD3^+^CD4^+^CD45RO^+^ and **b** CD3^+^CD8^+^CD45RO^+^ T cells. (**c**) IFNγ- and IL4-producing cells within the CD4^+^ T cell population, in CD4^+^ (open symbols) or CD3^+^ (closed symbols) T cell cultures upon O/N restimulation with PMA and ionomycin in absence of AKT-inhibitors, corrected for non-stimulated conditions. **d**–**h** Cells were restimulated in absence of AKT-inhibition for 7 days, after which degranulation and cytokine production was analyzed after O/N stimulation. **d** Fold expansion of CD8^+^ T cells between day 10 and 17 upon rechallenge with CD3/CD28 Dynabeads. **e** Percentages CD107a^+^CD3^+^CD8^+^ T cells. **f**–**i** Percentages IFNγ-, IL2-, and TNFα-producing within the CD8^+^ T cell population, **f** analyzed using SPICE software: the pie arcs depict the proportion of cells that produces a specific cytokine and pie slices the proportion of cells co-producing 0 to 3 different cytokines. **g** Total IFNγ-, IL2-, TNFα-, **h** double and i triple-producing CD3^+^CD8^+^ T cells in CD8^+^ (open symbols) or CD3^+^ (closed symbols) T cell cultures. Data of 4 independent donors is depicted as **a**, **b** **d**, **e** mean + SEM or as **c**, **g**–**i** mean with unique symbols. Statistical analysis was performed using Repeated Measures ANOVA followed by Bonferroni’s Multiple Comparison Test, comparing AKT-inhibited with Ctrl T cells within each starting population and **c**, **g**–**i** comparing cell composition within Ctrl or AKT-inhibited condition, or **a**–**d** Ctrl conditions were compared with a two-tailed paired *T* test. ***p < 0.001, **p < 0.01, *p < 0.05

As most T cell therapies are generated using polyclonal stimulation, AKT-inhibition was also investigated in combination with T cell expansion upon anti-CD3/CD28 bead stimulation instead of allogeneic-DCs. For polyclonal expanded cells, the cell composition had less effect on memory differentiation and subset skewing than in allogeneic DC cultures (Fig. 6a–c). Moreover, the effects of AKT-inhibition on CD62L expression were less prominent, however could preserve expression of CCR7 and CXCR4 indicating an early memory phenotype of both CD4^+^ and CD8^+^ T cells (Fig. 6a, b). Similarly, also here a change in cytokine production was observed with less IFNγ-producing and more IL4-producing CD4^+^ T cells (Fig. 6c). In contrast to allogeneic-DC expanded cultures, as cell composition had minimal influence in these assays, the reduction in IFNγ-producing CD4^+^ T cells was also observed in CD3^+^ T cell cultures. Importantly, rechallenge experiments showed clearly enhanced expansion capacity of AKT-inhibited CD8^+^ T cells cultures, but only in single CD8^+^ T cell cultures (Fig. 6d). Similar to allogeneic DC cultures, this could not be observed in CD3^+^ T cell cultures were expansion of non-AKT-inhibited CD8^+^ T cells was already higher, most likely due to the help by CD4^+^ T cells (Fig. 6d). Combined, also in polyclonal-expanded T cell cultures, AKT-inhibition preserved the early memory phenotype of CD4^+^ and CD8^+^ T cells and skewed CD4^+^ T cells toward Th2 at the expense of a Th1 profile, with limited influence of cell composition. However, the rechallenge expansion capacity of CD8^+^ T cells in the presence of AKT-inhibited CD4^+^ T cells could not be further enhanced using AKT-inhibition.

Also for the polyclonal expanded CD8^+^ T cells, we analyzed degranulation and polyfunctional effector cytokine production after one week of rechallenge in absence of AKT-inhibitors. In contrast to allogeneic-expanded CD8^+^ T cells, no effect on degranulation capacity was seen for AKT-inhibition (Fig. 6e). Furthermore, while in allogeneic expanded cultures an increase was observed for cytokine production, here IL2 and TNFα production was not affected, and production of IFNγ was even reduced in polyclonal-expanded AKT-inhibited CD8^+^ T cells (Fig. 6f–l). Combined this resulted in a reduced polyfunctionality for AKT-inhibited CD8^+^ T cells. Altogether, these data demonstrate that the beneficial effect of AKT-inhibition on the degranulation and polyfunctionality of CD8^+^ T cells is dependent on the composition of the cell starting material and expansion strategy, where the presence of CD4^+^ T cells as well as polyclonal stimulation impede the favorable effect of AKT-inhibition on CD8^+^ T cells.

## Discussion

Adoptive T cell therapy is a promising treatment for hematological malignancies as shown by impressive responses of anti-CD19 CAR T cells in patients with acute lymphoblastic leukemia and B cell lymphoma [37–39]. However, in other malignancies like chronic lymphocytic leukemia complete response rates with CAR-T cells are lower, because of early loss of tumor-reactive T cell persistence [5, 39, 40]. Importantly, complete responses have been associated with robust T cell expansion and persistence, and an early memory phenotype of the infused T cells [5, 19, 37, 41–43]. Therefore, efficacy of adoptive T cell therapy could be improved by infusion of tumor-reactive T cells with early memory traits. Previously, we and others showed that inhibition of the AKT-pathway is a powerful means to generate these stem cell memory-like CD8^+^ T cells ex vivo. [7–9, 11] Furthermore, AKT-inhibition did not harm the expansion of minor histocompatibility antigen-specific CD8^+^ T cells from the naive repertoire for post-allogeneic stem cell transplantation immunotherapy [11]. Hereto, it would be interesting to apply AKT-inhibition to manufacturing protocols of adoptive T cell therapeutics. However, when generating or expanding tumor-reactive T cells for CAR T cell, TCR-transduced T cell or TIL therapy, PBMCs or total CD3^+^ T cells are mostly used instead of purified CD8^+^ T cells [2, 37–40, 44]. This can be an advantage because of the supportive role of CD4^+^ T helper cells for CD8^+^ T cells [16–18]. However, as the effect of ex vivo AKT-inhibition on CD4^+^ T cells is not well studied, we here aimed to investigate the effect of transient AKT-inhibition on CD4^+^ T cell differentiation and functionality, and their effect on CD8^+^ T cell function.

In agreement to our previous finding for CD8^+^ T cells, both AktiVIII and GDC preserved the CD4^+^ early memory T cell phenotype, showing higher expression of CCR7, CD62L and CXCR4, while allowing T cell proliferation. This would improve the therapeutic potency as mouse studies revealed a superior anti-tumor effect of early memory CD4^+^ T cells [19]. Anti-CD19 CAR T cells generated from CD4^+^ T_N_ and T_CM_ cells exhibited a higher expression of CD62L and had favorable effects on survival of tumor-bearing mice compared to treatment with CAR T cells generated from CD4^+^ T_EM_. Whether therapeutic potency would be improved by superior support to other killer immune cells, or whether AKT-inhibited CD4 T cells could be cytotoxic precursors themselves requires more analysis [45]. Together this indicates that the preserved memory phenotype of AKT-inhibited CD4^+^ T cells could by itself be beneficial for adoptive T cell therapy efficacy.

However, besides the favorable effect on the CD4^+^ T cell memory state, we observed that ex vivo AKT-inhibition also modulated the CD4^+^ Th-subset skewing. We demonstrated that AKT-inhibition during T cell expansion reduced Th1-characterisitics, while promoting Th2 differentiation, especially when focusing on cytokine production of T cells. Previous studies showing that stimulation of AKT-signaling promotes Th1 skewing, matches our observation of less Th1/IFNγ-producing cells by inhibition of AKT-signaling [15, 46, 47]. While the increased Th2 differentiation by AKT-inhibition has not been described previously, it is known that Th1 and Th2 cells could potentially inhibit each other via production of IFNγ and IL4, respectively [48]. Therefore, less Th1-produced IFNγ in AKT-inhibited compared to control cultures might result in reduced inhibition of Th2 differentiation, resulting in higher numbers of Th2 cells. Together, this demonstrates that while AKT-inhibition might be beneficial by preserving early memory CD4^+^ T cells, one should be aware on the collateral, and possibly undesirable, effects on Th-subset skewing.

Since therapeutic T cell products are most often generated from PBMCs or CD3^+^ T cell fractions, which contain besides CD4^+^ T_N_ cells also further differentiated CD4^+^ as well as CD8^+^ T cells, we evaluated the effect of AKT-inhibition on CD4^+^ T cells starting from either total CD4^+^ or CD3^+^ T cell cultures. When starting the cultures with total CD4^+^ T cells instead of CD4^+^ T_N_ cells alone, the effects on CD4^+^ Th-subset skewing were less pronounced. This is probably due to the difference in skewing-potential of naive versus effector and memory subsets [49]. Similarly, when cultures were started with CD3^+^ T cells, the CD8^+^ T cells most likely affected (AKT-inhibition of) CD4^+^ T cells. Presence of CD8^+^ T cells reduced IFNγ-production of (non-AKT-inhibited) control CD4^+^ T cells. As a consequence, addition of an AKT-inhibitor did no longer reduce the number of IFNγ-producing CD4^+^ T cells. Additionally, CD8^+^ T cells also affected the preservation of CD62L expression on (AKT-inhibited) CD4^+^ T cells. Overall, reduced memory differentiation was observed in combined (CD4^+^ plus CD8^+^) T cell cultures compared to single cultures. Moreover, where TCR strength can control the differentiation into effector and memory cells, the effect of AKT-inhibition is most likely also dependent on the balance between AKT-inhibition and the magnitude of T cell stimulation [50]. Furthermore, it has been described that CD8^+^ effector T cells can boost both phenotypic and functional differentiation of naive T cells via non-apoptotic FAS signaling [51]. The possible cell interaction and cytokines produced by CD8^+^ T cells in CD3^+^ T cell cultures might therefore result in stronger T cell stimulation, and as a consequence smaller effects of AKT-inhibition on CD4^+^ effector memory and Th-differentiation. Taken together, the cell composition of the culture and the several factors produced or consumed by these CD4^+^ and CD8^+^ T cells could influence the balance of stimulation and thereby (the effect of AKT-inhibition on) their counterparts.

Finally, in our study we used both DCs and polyclonal stimulation for T cell expansion. Though DCs are the most physiological activation, most approaches employ polyclonal stimulation for the generation of therapeutic T cell products. Both strategies resulted in preserved early memory differentiation and a change in CD4^+^ Th-cytokine production. However, when using polyclonal stimulation, CD62L expression was less preserved, and less IFNγ-producing CD4^+^ and CD8^+^ T cells were observed upon AKT-inhibition, indicating crucial differences between the final T cell products. Additionally, AKT-inhibition did not improve the degranulation-potency of CD8^+^ T cells in polyclonal expanded cultures, which was observed upon expansion by DCs. Though the degranulation and cytokine production capacity cannot be compared directly between expansion strategies due to different read-out methods, the lack of favorable effect of AKT-inhibition within the polyclonal expansion model is disturbing.

As is described for CD4^+^ T helper cells, we observed a supportive role of CD4^+^ T cells to CD8^+^ T cell functionality in control cultures [16–18]. In DC stimulated cultures, the presence of CD4^+^ T cells in control conditions resulted in a better rechallenge capacity, degranulation and more polyfunctionality of non-AKT-inhibited CD8^+^ T cells. This supportive role, based on cytokine support and a positive balance in co-stimulatory and co-inhibitory molecule expression provides rational for adoptive T cell therapies containing both CD4^+^ and CD8^+^ T cells [16]. Importantly, AKT-inhibition in these cultures preserved the early memory CD8^+^ T cell phenotype and resulted in a further increased CD8^+^ T cell expansion upon antigen recall both in the absence and presence of CD4^+^ T cells. However, AKT-inhibition could no longer increase the degranulation or polyfunctionality of CD8^+^ T cells when they had been expanded in the presence of AKT-inhibited CD4^+^ T cells, questioning the application of AKT-inhibition for combined T cells cultures. Potentially, single expanded AKT-inhibited CD8^+^ T cells could perform even better, as in these read-out assays they are were not supported by any CD4^+^ T cells. (Non-inhibited) CD4^+^ T cells could enhance the functionality of these single expanded AKT-inhibited CD8^+^ T cells. Overall, clinical application of AKT-inhibition could be promising and other studies have shown that AKT and PI3K-inhibition is feasible during CAR transduction, where it preserves CD62L expression on CAR T cells [9, 12, 15]. With this strategy, pharmaceutical inhibitors are solely applied during ex vivo expansion, and are not present in vivo as it is washed away in the therapeutic product before infusion. Most studies where AKT- or PI3K-inhibition was applied during the generation of CAR T cells, focused on effects on CD8^+^ T cells and only minor attention was given to modulation of CD4^+^ T cells following PI3K/AKT-pathway inhibition [9, 15]. However, Petersen et al. generated anti-CD5 CAR T cells in the presence of a PI3K-inhibitor and analyzed cytokine profiles of CD4^+^ and CD8^+^ T cells separately [15]. They showed a trend toward less IFNγ-producing cells for both cell types, similar to our observations. Moreover, Urak et al. applied AKT-inhibition for the generation of anti-CD19 CAR T cells and showed retained CD62L expression [12]. In mouse models, a combination of these AKT-inhibited CD4^+^ and CD8^+^ CAR T cells showed better anti-tumor effects compared to control CAR T cells. However, controls were missing to conclude whether this was due to improved CD4^+^ or CD8^+^ T cells, or whether AKT-inhibited CD8^+^ CAR T cells with non-inhibited CD4^+^ CAR T cells would have been even better. Considering our results, future research could focus on a separated T cell expansion strategy, where the most favorable (AKT-inhibited) conditions could be explored for CD4^+^ and CD8^+^ T cells without effecting each other.

Since ex vivo AKT-inhibition during T cell expansion, facilitates the generation of early memory T cells with a T_SCM_-like phenotype, it is a promising strategy to apply in the generation of CAR T cells, TCR-transduced T cells and TIL products. However, despite the observed favorable effects, one should be aware on the choice of expansion strategy and starting cell composition. Our data show effects of AKT-inhibition on CD4^+^ Th-differentiation, which when cultured together could have a negative influence on AKT-inhibited CD8^+^ T cell rechallenge capacity and polyfunctionality. Since an early memory differentiation status and polyfunctional characteristics of CD8^+^ T cells are postulated to be pivotal for therapeutic efficacy, we recommend to carefully determine the optimal expansion method. Where possible, cell-based expansion should be considered. Moreover, AKT-inhibited CD8^+^ T cells should be expanded in the absence of CD4^+^ T cells, where CD4^+^ T cells could be expanded separately with or without AKT-inhibition, followed by co-infusion for therapy of cancer patients.

### Electronic supplementary material

Below is the link to the electronic supplementary material.

Supplementary file1 (PDF 1058 kb)
