# Supplementary material for: Cell composition and expansion strategy can reduce the beneficial effect of AKT-inhibition on functionality of CD8+ T cells
Source: Cancer Immunol Immunother. 2020 Jun 5;69(11):2259–73. doi: 10.1007/s00262-020-02612-w (PMC7568704; doi:10.1007/s00262-020-02612-w)
Supplement: Supplementary file 1 — Supplementary file1 (PDF 1058 kb) [file 262_2020_2612_MOESM1_ESM.pdf]

Supplementary Figure 1

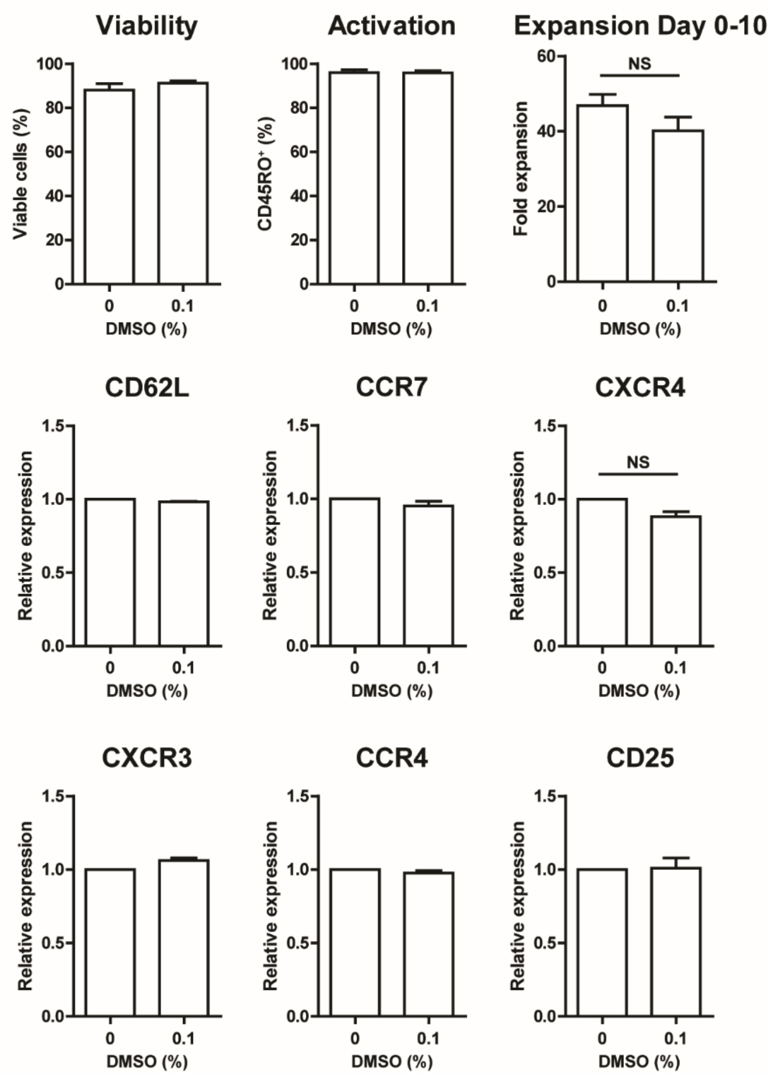

Supplementary Figure 1 DMSO has no effect on viability, activation, expansion and differentiation of CD4+ T cells

CD4+ T_N cells were stimulated with DCs in absence or presence of 0.1% DMSO and analyzed on day 10 for viability, activation, expansion, memory (CD62L, CCR7, CXCR4) and T cell subset-associated (CXCR3, CCR4, CD25) phenotype. Mean + SEM of 2 independent donors. Statistical analysis was performed using a two-tailed unpaired T test, no significant changes were found.

Supplementary Figure 2

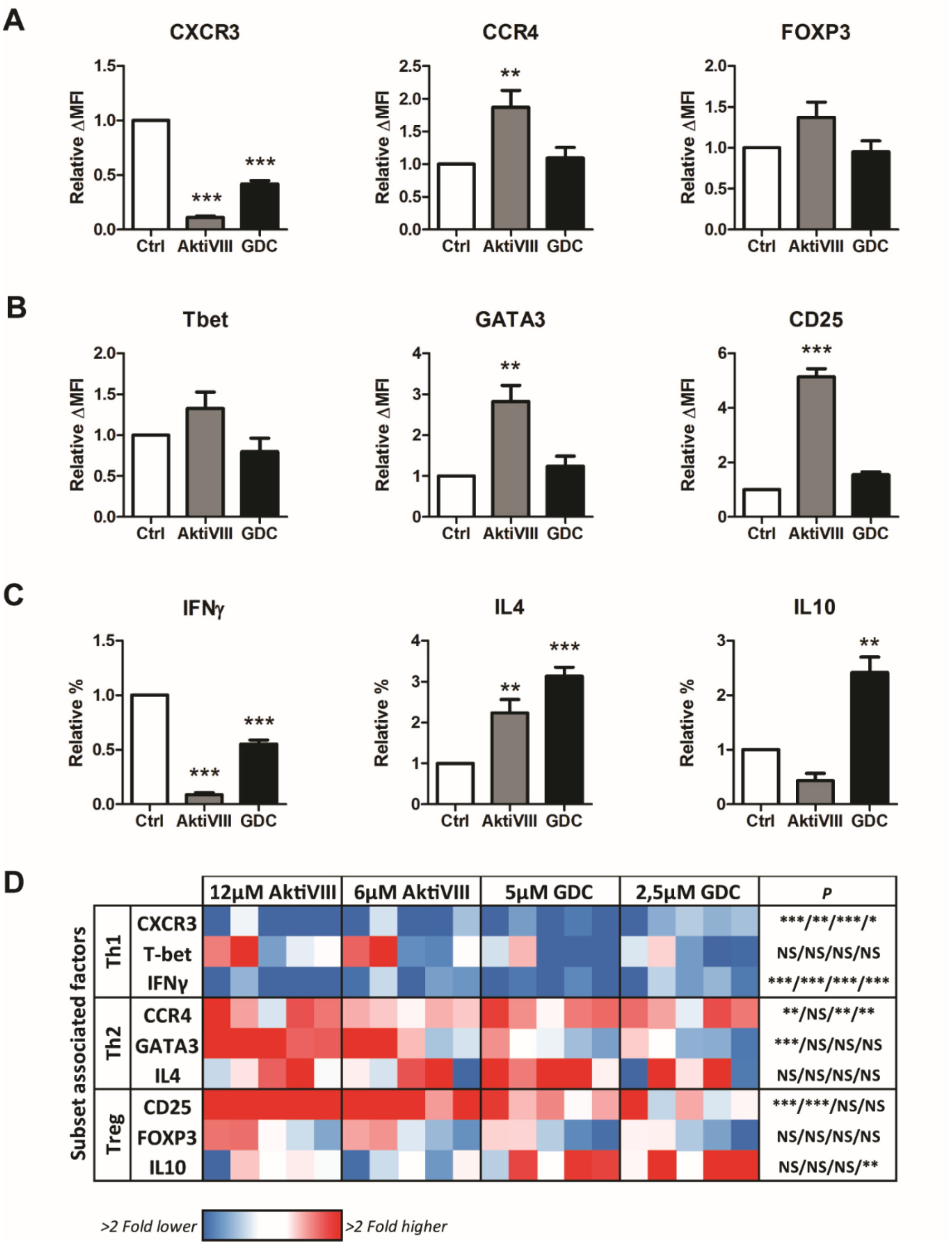

Supplementary Figure 2 AKT-inhibition modulates $CD4^+$ T helper cell skewing

$CD4^+$ T_N cells were expanded with allogeneic dendritic cells in presence of DMSO (Ctrl), AktiVIII or GDC for 10 days. (**A-C**) Relative (**A**) surface expression, (**B**) intracellular transcription factor expression and (**C**) cytokine production of $CD3^+CD4^+CD45RO^+$ T cells, after O/N restimulation with allogeneic dendritic cells in absence of AKT-inhibitors, corresponding to data shown in heat-map (Figure 2D, 18μM AktiVIII

and 10µM GDC). Mean+SEM shown for 4-5 independent donors. (**D**) Heatmap showing fold change compared to Ctrl condition of different T-helper cell associated parameters for 4-5 independent donors using 12 or 6µM AktiVIII and 5 or 2.5µM GDC. Grey = no data. Cytokine production was corrected for non-stimulated conditions. Statistical analysis was performed using Repeated Measures ANOVA followed by Bonferroni's Multiple Comparison Test of AKT-inhibited versus Ctrl T cells. ***p<0.001, **p<0.01, *p<0.05.

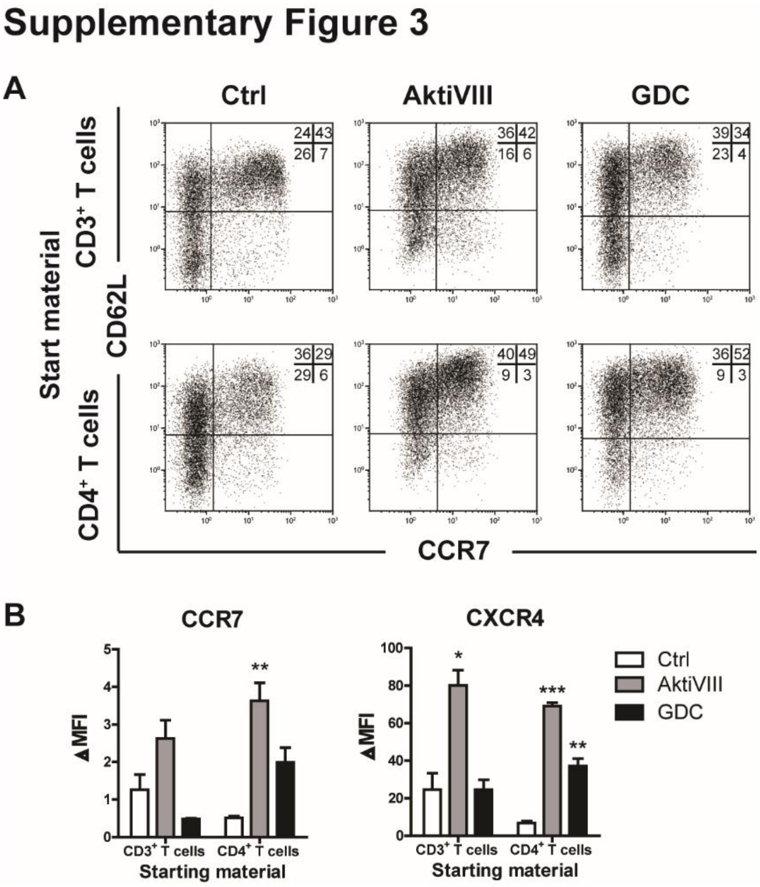

Supplementary Figure 3 Preserved memory differentiation of CD4+ T cells by AKT-inhibition in CD3+ T cell and CD4+ T cell cultures

CD3+ T cells or CD4+ T cells were stimulated with allogeneic dendritic cells in presence of DMSO (Ctrl), AktiVIII (18µM) or GDC (10µM). CD4+ T cell differentiation was analyzed on day 10. (**A**) Dotplots of CD62L and CCR7 expression gated on CD3+CD4+CD45RO+ T cells of 1 representative donor. Gates were placed based on background staining, numbers represent percentage gated cells. (**B**) CCR7 and CXCR4 expression of 1 representative donor out of 3 donors, Mean+SD, n=2. Statistical analysis was performed using One-way ANOVA followed by Bonferroni's Multiple Comparison Test of AKT-inhibited versus Ctrl T cells within each starting population. ***p<0.001, **p<0.01, *p<0.05.

Supplementary Figure 4

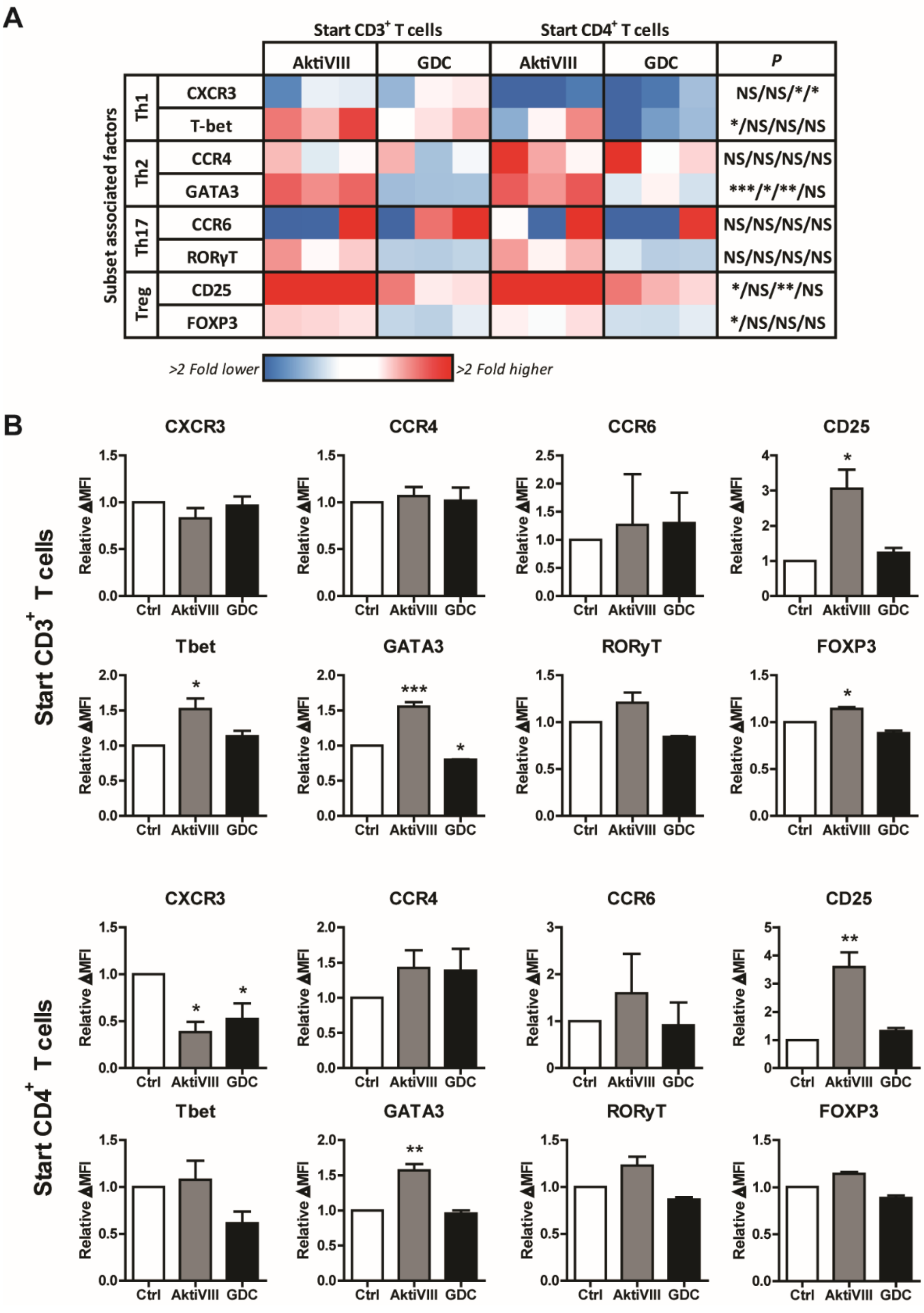

Supplementary Figure 4 AKT-inhibition does not impede Th1 skewing of CD4$^+$ T cells in cultures with total CD3$^+$ T cells

Total CD3$^+$ T cells or CD4$^+$ T cells were stimulated with allogeneic dendritic cells in presence of DMSO (Ctrl), AktiVIII (18µM) or GDC (10µM) for 10 days. (**A**) Heatmap and (**B**) statistical analysis showing fold change of expression of extracellular markers and transcription on AKT-inhibited compared to Ctrl conditions, gated on CD3$^+$CD4$^+$CD45RO$^+$ T cells within each starting material on day 10, mean+SEM of 3 independent donors. Statistical analysis was performed using Repeated Measures ANOVA followed by Bonferroni's Multiple Comparison Test of AKT-inhibited versus Ctrl T cells. ***p<0.001, **p<0.01, *p<0.05.

Supplemental Figure 5

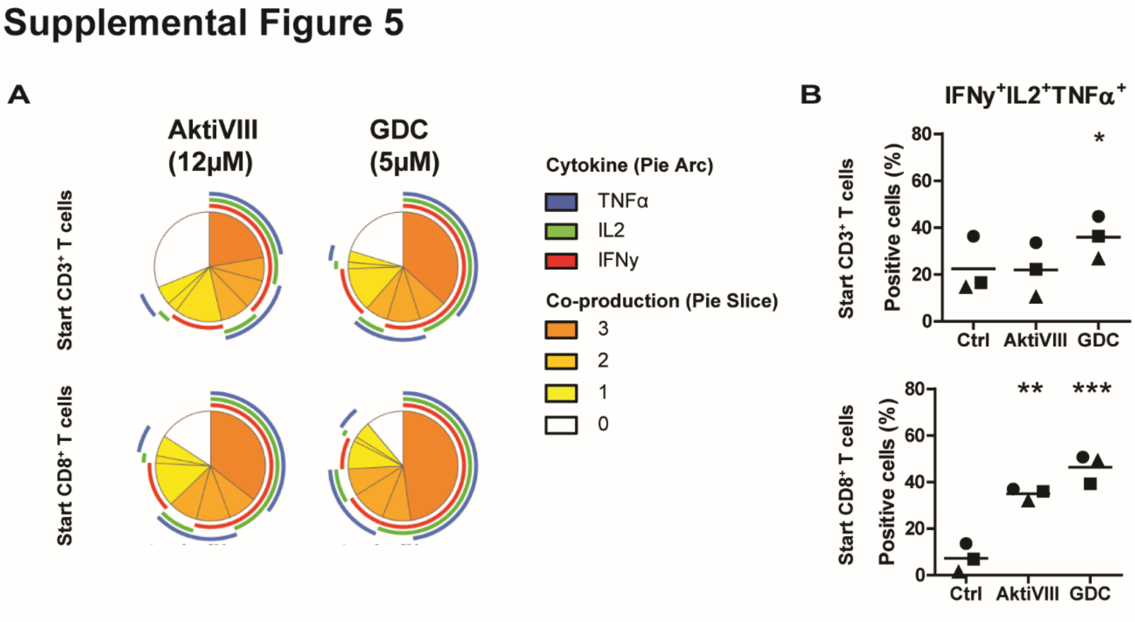

Supplementary Figure 5 AKT-inhibition of allogeneic dendritic cell expanded CD8+ T cells enhances polyfunctionality also at lower dosages

Total CD3+ T cells or CD8+ T cells were stimulated with allogeneic dendritic cells in presence of DMSO (Ctrl), AktiVIII (12 µM) or GDC (5µM) for 10 days. Cells were restimulated in absence of AKT-inhibition for 7 days, and cytokine production was analyzed after O/N allogeneic dendritic cell stimulation. (**A**) Percentages IFNγ-, IL2-, and TNFα-producing CD3+CD8+ T cells, analyzed using SPICE software. The pie arcs depict the proportion of cells that produces a specific cytokine and pie slices the proportion of cells co-producing 0 to 3 different cytokines. (**B**) Percentages IFNγ-, IL2-, TNFα-triple-producing CD3+CD8+ T cells. Statistical analysis was performed using Repeated Measures ANOVA followed by Bonferroni's Multiple Comparison Test of AKT-inhibited versus Ctrl T cells within each starting population. ****p*<0.001, ***p*<0.01, **p*<0.05.
